# The efficacy and safety of acupuncture in the treatment of erectile dysfunction: A protocol for systematic review and meta-analysis

**DOI:** 10.1097/MD.0000000000025892

**Published:** 2021-05-28

**Authors:** Yuliang Zhou, Shenghui Chen, Duanjun Zhang, Huiyu Lu, Wenliang Yao, Wanxue Jiang, Yinglv Yu, Chaoren Jiang

**Affiliations:** Nanchang Reproductive Hospital, Nanchang, China.

**Keywords:** acupuncture, complementary therapy, efficacy, erectile dysfunction, impotence, TCM

## Abstract

**Background:**

Erectile dysfunction (ED) can negatively affect men's mental health, interpersonal relationships, and overall well-being. ED has affected >150 million men worldwide, and this number will reach approximately 322 million by 2025. Although PDE5-Is is a landmark in the treatment of erectile dysfunction, it may have side effects such as penile pain, cardiovascular dysfunction, and deafness. Some studies have shown that acupuncture may have a positive effect on the pathophysiology of ED. Therefore, we will select all randomized controlled trials related to evaluate the efficacy and safety of acupuncture treatment of ED.

**Methods:**

This study will systematically search 7 digital databases including China National Knowledge Infrastructure, Wanfang, VIP, China Biology Medicine, Cochrane Library, PubMed, and Embase for randomized controlled trials without language restrictions. Two researchers will independently read the title, abstract, and full text to screen for studies that can be included in the meta-analysis. If there is any dispute, the third party will be required to reach a consensus.

**Results:**

The purpose of this study is to evaluate the efficacy and safety of acupuncture in the treatment of ED and the difference in the impact of different types of acupuncture on ED.

**Conclusion:**

Judge whether acupuncture and moxibustion can help improve the symptoms of ED by evaluating relevant literatures, and make up for the lack of relevant research.

**INPLASY registration number:**

INPLASY 202140040

## 1 Introduction

Erectile dysfunction (ED) is a male disease characterized by difficulty in erecting or maintaining sufficient erection strength for satisfactory sexual performance.^[1]^ Penile erection is accomplished by the regulation of psychological factors and hormonal status.^[2,3]^ Risk factors for erectile dysfunction include smoking,^[4]^ alcohol,^[5]^ diabetes,^[6]^ depression,^[7]^ metabolic syndrome, and obesity.^[8,9]^ ED can negatively affect men's mental health, interpersonal relationships, and overall well-being.^[10]^ Erectile dysfunction has affected >150 million men worldwide, and by 2025, this number will reach approximately 322 million.^[11]^ Ample evidence show that ED is a risk marker for treatable underlying diseases. And if ED is not effectively treated, these diseases will reduce the quality and length of life.^[12]^ In general, oral PDE5-Is is the main drug for the treatment of erectile dysfunction. Other treatments include lifestyle changes, injection therapies, testosterone therapy, penile devices, and psychotherapy.^[13–15]^ Oral PDE inhibitors are a convenient, effective, and widely available treatment option for erectile dysfunction.^[16]^ Although PDE5-Is is a landmark in the treatment of erectile dysfunction, they are far from perfect. As we all know, the disadvantages of PDE5-Is are the uncommon success rate, absence of spontaneity, and life-long drug commitment. In addition, these therapies are not always effective and may have side effects such as penile pain, cardiovascular dysfunction, and deafness.^[17,18]^ Currently, specific treatments not only for PDE5 enzyme inhibition are under development.^[19]^

Acupuncture is a component of Traditional Chinese Medicine that can be traced back at least 2500 years. Some studies have shown that the neurophysiological effects of acupuncture, due to central nervous system activation and neurotransmitter regulation, may positively affect the pathophysiology of ED.^[20,21]^ In urinary system diseases, acupuncture has been successful as the main or adjuvant therapy for enuresis,^[22]^ bladder instability,^[23]^ and male infertility.^[24]^ Through preliminary retrieval and analysis of database resources, we found that randomized controlled trials (RCTs) of acupuncture treatment in the ED are gradually increasing. Therefore, the purpose of our program is to select all RCTs related to evaluate the efficacy and safety of acupuncture in the treatment of ED in comparison with other drug therapies.

## 2 Objectives

The aims are to compare the efficacy of acupuncture for the treatment of ED and to analyze the curative effect of different acupuncture interventions on ED.

## 3 Methods and analysis

### 3.1 Study registration

The protocol of our study is conducted in strict accordance with the Preferred Reporting Items for Systematic Reviews and Meta-analysis Protocols guidelines and the Cochrane Handbook.^[25,26]^ This protocol has been registered on INPLASY (registration number: INPLASY 202140040: https://inplasy.com/inplasy-2021-4-0040/).

### 3.2 Inclusion criteria

#### 3.2.1 Type of studies

Only RCTs will be included.

#### 3.2.2 Type of participants

Participants are patients >18 years of age who are diagnosed with ED. ED must be diagnosed by clinical and/or instrumental methods. The diagnosis will be based on the Diagnostic and Statistical Manual of Mental Disorders Third Edition (DSM)-III, DSM-III-R, DSM-IV, International Statistical Classification of Diseases and Related Health Problems (ICD)-10 criteria or any other described criteria.

#### 3.2.3 Type of interventions

The types of acupuncture in the intervention group include: manual acupuncture, electric acupuncture, scalp acupuncture, ear acupuncture, moxibustion, and fire needling.

#### 3.2.4 Type of comparators

Control measures can include: placebo/sham acupuncture or other interventions (such as drugs, physical therapy).

#### 3.2.5 Types of outcome measures

##### 3.2.5.1 Primary outcomes

The main result will be an improvement in sexual activity. This will be assessed through validated questionnaires such as the International Index of Erectile Function.

##### 3.2.5.2 Secondary outcomes

Secondary outcome measures include quality of Life, improvement in anxiety or depression scales, and the rate of adverse effects.

### 3.3 Exclusion criteria

Exclusion criteria are: non-RCTs; trials using unverified questionnaires or without a clear description of the evaluation method; studies with imbalanced or incomparable baseline data between the 2 groups will be excluded.

### 3.4 Search methods for identification of studies

#### 3.4.1 Electronic searches

This study will systematically search 7 digital databases including China National Knowledge Infrastructure, Wanfang, VIP, China Biology Medicine, Cochrane Library, PubMed, and Embase for RCTs without language restrictions. The following search terms will be used individually or in combination: acupuncture, electro-acupuncture, Warm Acupuncture, elongated needle, erectile dysfunction, impotence, and ED. Two researchers will independently read the title, abstract, and full text to screen for studies that can be included in the meta-analysis. The searching strategy of PubMed is presented in Table 1.

**Table 1:** Search strategy used in PubMed database.

Order	Search items
#1	(((((((Erectile Dysfunction[MeSH Terms]) OR (Dysfunction, Erectile)) OR (Male Sexual Impotence)) OR (Impotence, Male Sexual)) OR (Sexual Impotence, Male)) OR (Male Impotence)) OR (Impotence, Male)) OR (Impotence)
#2	((((((((((((((((Acupuncture[MeSH Terms]) OR (Pharmacopuncture)) OR (Acupuncture Therapy)) OR (Electroacupuncture)) OR (Manual Acupuncture)) OR (Dry Needle)) OR ((Moxibustion[MeSH Terms]) OR (moxibustion))) OR (Acupuncture, Ear[MeSH Terms])) OR (acupuncture, Ear)) OR (ear acupuncture)) OR (Auricular Acupuncture)) OR (Ear Acupuncture)) OR (Acupuncture, Auricular)) OR (acupuncture, Auricular)) OR (auricular acupuncture)) OR (Warm Acupuncture)) OR (Elongated Needle)
#3	randomized controlled trial[Publication Type] OR randomized[Title/Abstract] OR placebo[Title/Abstract]
#4	#1 AND #2 AND #3

#### 3.4.2 Searching other resources

We will search the National Institutes of Health clinical registry Clinical Trials, International Clinical Trials Registry Platform, and ClinicalTrials.gov to find any ongoing or unpublished trial.

### 3.5 Selection of studies

**Figure 1:**
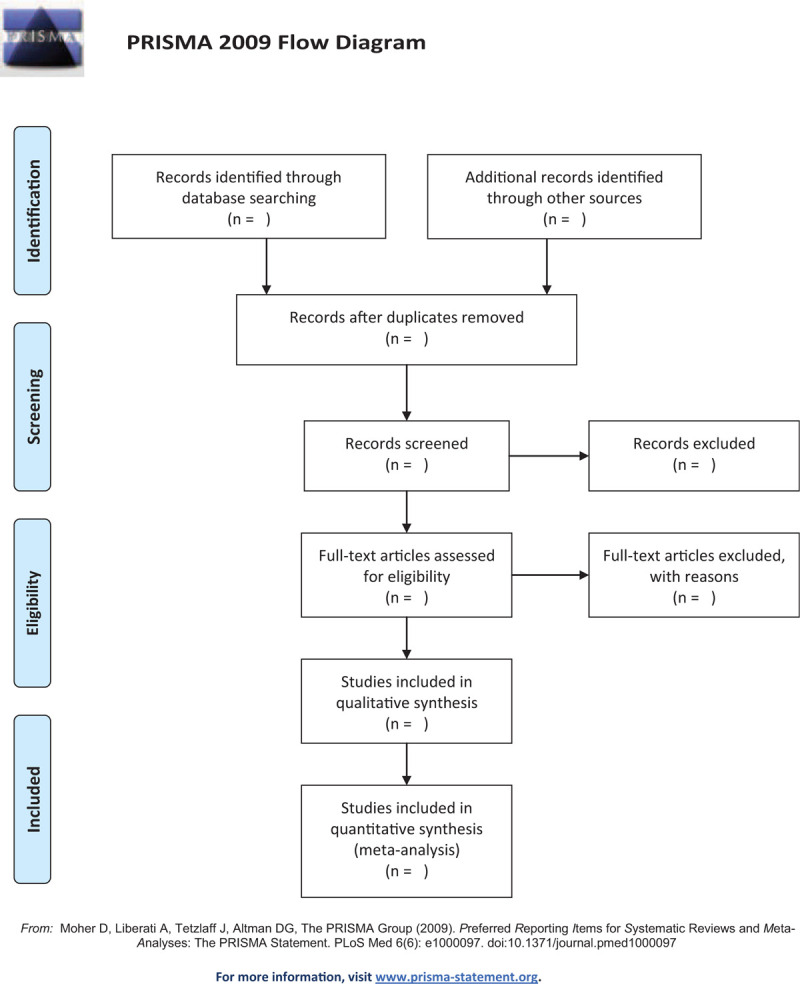
Flowchart of literature selection.

The studies of electronic searches will be exported to EndNote V.9.1 software to remove duplicate literature. Literature screening will be conducted independently by 2 reviewers according to the inclusion and exclusion criteria. They first read the title and abstract of the studies to exclude irrelevant trials. Then they read the full text to further screen out the documents that meet the requirements. Finally, valid data will be extracted in the included literature one by one. If there is a disagreement during the screening process, the final decision will be reached through discussion with the third reviewer. The selection process will be showed in a PRISMA flow diagram (Fig. 1).

### 3.6 Data extraction and management

Two authors will independently select the trials included in the review according to the inclusion/exclusion criteria. Any disagreement will be resolved by discussion. We will perform data extraction using a Microsoft Excel spreadsheet. Information extracted from each included article will include first author, publication year, sample size, characteristics of participants, type of treatments, and outcome measures. If the necessary data are not available in the trial reports, further information will be sought by contacting corresponding author.

### 3.7 Assessment of the methodological quality

The risk of bias in the included literature will be assessed according to the Cochrane Collaboration's tool for assessing risk of bias.^[26]^ We will assess the risk of bias from the following 7 items: random sequence generation, allocation concealment, blinding of participants and personnel, blinding of outcome assessment, incomplete outcome data, selective reporting, and other sources of bias. The risk of bias graph and the risk of bias summary will be generated by Review Manager (RevMan) V.5.3 software. Any disagreement should be solved in consultation with a third reviewer.

### 3.8 Measures of treatment effect

For continuous data, the mean difference will be used in the analysis of continuous outcomes with 95% confidence intervals. In case outcome variables have different scales, the standardized mean difference will be used with 95% confidence intervals. The relative risk will be used to assess dichotomous outcomes.

### 3.9 Dealing with missing data

We will attempt to contact authors to obtain missing data. If we cannot contact the original authors, the studies will be excluded from the data synthesis.

### 3.10 Assessment of heterogeneity

The Cochran *Q* statistics will be employed to assess heterogeneity. If there is significant clinical heterogeneity or methodological heterogeneity (*P* < .1, I^2^ >50%), the subgroup analysis will be performed to explore sources of heterogeneity. To assess potential bias resulting from baseline risk, we will perform meta-regression with regressors which included age of participants, sample size, duration of disease, course of treatment, and so on. Besides, sensitivity analyses will be performed by excluding studies with a high risk of bias or poor quality to judge the stability of the results.

### 3.11 Data synthesis

Fixed-effects models will be used if the *I*^2^ value is <50%. Otherwise, we will remove low-quality studies and use sensitivity analysis to investigate which study has the most significant impact on heterogeneity. If quantitative synthesis is not possible, we will make a qualitative description.

### 3.12 Subgroup analysis

If there is significant heterogeneity between the study results, we will perform a subgroup analysis to investigate differences in sex, age, types of acupuncture interventions styles, and so on.

### 3.13 Sensitivity analysis

Sensitivity analysis will be conducted to explore the effects of trial risk of bias on important outcomes. Several factors in the meta-analysis process will be taken into consideration, such as low-quality research, small sample research, and so on. In addition, we will give the results of the sensitivity analysis in the summary table. The results of the sensitivity analysis will discuss the risk of bias in the meta-analysis.

### 3.14 Grading the quality of evidence

Two reviewers will independently use the Grading of Recommendations Assessment, Development and Evaluation. According to the grading standard, the quality of evidence is graded as high, medium, low, or very low.^[27]^

### 3.15 Ethics and dissemination

The study will be published in a peer-reviewed journal or relevant conference. No ethical approval is required. The results of the study will provide potential evidence in advancing the therapeutic strategy of patients with ED.

## 4 Discussion

Epidemiological studies have found that the prevalence of ED is high. As the life expectancy of the general population increases, the health care burden and quality of life issues associated with ED are expected to be considerable. The current treatments used for ED include oral drug therapy, intracavernosal injection and implantation of penile prostheses. PDE5-Is is currently used as a first-line treatment for ED. However, there are still a large number of patients who do not respond to this therapy. In addition, implanting penile prostheses is expensive and may cause penile deformities. Therefore, an ideal ED treatment method has not yet been developed. With the increasing use of acupuncture in the treatment of ED, our systematic review aims to evaluate the current evidence of the effectiveness and safety of acupuncture in the treatment of ED and provide other treatment options for ED patients.

## Author contributions

**Conceptualization:** Yuliang Zhou, Shenghui Chen.

**Data curation:** Yuliang Zhou, Duanjun Zhang, Yinglv Yu.

**Formal analysis:** Wanxue Jiang, Wengliang Yao, Huiyu Lu.

**Methodology:** Yinglv Yu, Chaoren Jiang, Wenliang Yao.

**Software:** Duanjun Zhang, Huiyu Lu, Wanxue Jiang.

**Supervision:** Shenghui Chen, Duanjun Zhang.

**Writing – original draft:** Yuliang Zhou, Duanjun Zhang, Yinglv Yu.

**Writing – review & editing:** Huiyu Lu, Wanxue Jiang, Shenghui Chen.

## References

[1] Najari BB Kashanian JA. Erectile dysfunction. JAMA 2016;316:1838. 27802547 10.1001/jama.2016.12284

[2] Mitidieri E Cirino G d’Emmanuele di Villa Bianca R. Pharmacology and perspectives in erectile dysfunction in man. Pharmacol Ther 2020;208:107493. 31991196 10.1016/j.pharmthera.2020.107493

[3] Gratzke C Angulo J Chitaley K. Anatomy, physiology, and pathophysiology of erectile dysfunction. J Sex Med 2010;7 (1 pt 2):445–75. 20092448 10.1111/j.1743-6109.2009.01624.x

[4] Ponholzer A Temml C Mock K. Prevalence and risk factors for erectile dysfunction in 2869 men using a validated questionnaire. Eur Urol 2005;47:80–6. 15582253 10.1016/j.eururo.2004.08.017

[5] Wang XM Bai YJ Yang YB. Alcohol intake and risk of erectile dysfunction: a dose-response meta-analysis of observational studies. Int J Impot Res 2018;30:342–51. 30232467 10.1038/s41443-018-0022-x

[6] Binmoammar TA Hassounah S Alsaad S. The impact of poor glycaemic control on the prevalence of erectile dysfunction in men with type 2 diabetes mellitus: a systematic review. JRSM Open 2016;7:2054270415622602. 26981254 10.1177/2054270415622602 PMC4776250

[7] Liu Q Zhang Y Wang J. Erectile dysfunction and depression: a systematic review and meta-analysis. J Sex Med 2018;15:1073–82. 29960891 10.1016/j.jsxm.2018.05.016

[8] Esposito K Giugliano D. Obesity, the metabolic syndrome, and sexual dysfunction in men. Clin PharmacolTher 2011;90:169–73. 10.1038/clpt.2011.91 21613988

[9] Alberti KG Eckel RH Grundy SM. Harmonizing the metabolic syndrome: a joint interim statement of the International Diabetes Federation Task Force on Epidemiology and Prevention; National Heart, Lung, and Blood Institute; American Heart Association; World Heart Federation; International Atherosclerosis Society; and International Association for the Study of Obesity. Circulation 2009;120:1640–5. 19805654 10.1161/CIRCULATIONAHA.109.192644

[10] Burnett AL Nehra A Breau RH. Erectile dysfunction: AUA Guideline. J Urol 2018;200:633–41. 29746858 10.1016/j.juro.2018.05.004

[11] Ayta IA McKinlay JB Krane RJ. The likely worldwide increase in erectile dysfunction between 1995 and 2025 and some possible policy consequences. BJU Int 1999;84:50–6. 10444124 10.1046/j.1464-410x.1999.00142.x

[12] Saigal CS Wessells H Pace J. Predictors and prevalence of erectile dysfunction in a racially diverse population. Arch Intern Med 2006;166:207–12. 16432090 10.1001/archinte.166.2.207

[13] Rosen R Goldstein I Heiman J. The process of care model for evaluation and treatment of erectile dysfunction. The Process of Care Consensus Panel. Int J Impot Res 1999;11:59–70. 10356665 10.1038/sj.ijir.3900411

[14] Hatzichristou D Rosen RC Derogatis LR. Recommendations for the clinical evaluation of men and women with sexual dysfunction. J Sex Med 2010;7 (1 pt 2):337–48. 20092443 10.1111/j.1743-6109.2009.01619.x

[15] Padma-Nathan H Christ G Adaikan G. Pharmacotherapy for erectile dysfunction. J Sex Med 2004;1:128–40. 16422967 10.1111/j.1743-6109.2004.04021.x

[16] Muneer A Kalsi J Nazareth I. Erectile dysfunction. BMJ (Clinical research ed) 2014;348:g129. 10.1136/bmj.g129 24468580

[17] Tharyan P Gopalakrishanan G. Erectile dysfunction. Clin Evid 2006;1227–51. 16973050

[18] Lowe G Costabile RA. 10-Year analysis of adverse event reports to the Food and Drug Administration for phosphodiesterase type-5 inhibitors. J Sex Meds 2012;9:265–70. 10.1111/j.1743-6109.2011.02537.x 22023666

[19] Shamloul R Ghanem H. Erectile dysfunction. Lancet (London, England) 2013;381:153–65. 10.1016/S0140-6736(12)60520-0 23040455

[20] Cheng KJ. Neuroanatomical basis of acupuncture treatment for some common illnesses. Acupunct Med 2009;27:61–4. 19502461 10.1136/aim.2009.000455

[21] Napadow V Ahn A Longhurst J. The status and future of acupuncture mechanism research. J Altern Complement Med (New York, NY) 2008;14:861–9. 10.1089/acm.2008.SAR-3 PMC3155097 18803495

[22] Yang C Hao Z Zhang LL. Efficacy and safety of acupuncture in children: an overview of systematic reviews. Pediatr Res 2015;78:112–9. 25950453 10.1038/pr.2015.91

[23] Mak TC Chen HY Cho WC. Acupuncture for overactive bladder in adults: a systematic review and meta-analysis. Acupunct Med 2019;37:321–31. 31433197 10.1136/acupmed-2017-011528

[24] Yao DF Mills JN. Male infertility: lifestyle factors and holistic, complementary, and alternative therapies. Asian J Androl 2016;18:410–8. 26952957 10.4103/1008-682X.175779 PMC4854092

[25] Shamseer L Moher D Clarke M. Preferred reporting items for systematic review and meta-analysis protocols (PRISMA-P) 2015: elaboration and explanation. BMJ (Clinical research ed) 2015;350:g7647. 10.1136/bmj.g7647 25555855

[26] Higgins JP Altman DG Gøtzsche PC. The Cochrane Collaboration's tool for assessing risk of bias in randomised trials. BMJ (Clinical research ed) 2011;343:d5928. 10.1136/bmj.d5928 PMC3196245 22008217

[27] Guyatt G Oxman AD Akl EA. GRADE guidelines: 1. Introduction-GRADE evidence profiles and summary of findings tables. J Clin Epidemiol 2011;64:383–94. 21195583 10.1016/j.jclinepi.2010.04.026

